# A Noteworthy Case Report of Neuroborreliosis in an Unvaccinated Pediatric Patient

**DOI:** 10.5811/cpcem.2020.9.48688

**Published:** 2020-10-26

**Authors:** Amber R. Walker, Teresita Morales-Yurick

**Affiliations:** Doctors Hospital, Department of Emergency Medicine, Columbus, Ohio

**Keywords:** Lyme disease, neuroborreliosis, Lyme meningitis

## Abstract

**Introduction:**

Lyme disease typically presents with viral-like symptoms and a pathognomonic rash. With disease progression, symptoms of nervous system involvement usually include facial nerve palsy and meningitis, but other atypical neurologic manifestations have less commonly been documented.

**Case Report:**

A six-year-old male presented with prolonged fevers, rash, headache, and non-specific neurologic symptoms. The diagnosis of neuroborreliosis with meningitis and polyradiculitis was confirmed with laboratory evaluation and lumbar puncture.

**Conclusion:**

Neuroborreliosis is a disseminated form of Lyme disease. While meningitis is a common sign, the presentation of polyradiculitis in children is rare and can lead to misdiagnosis and delay in treatment.

## INTRODUCTION

Lyme disease is the most common tick-borne disease in the United States.^1^ The disease is easily treatable once recognized; however, in children this diagnosis can be confounded by the similarity of the early symptoms of infection to other childhood illnesses.^2^ We present a case of a six-year-old-male who was evaluated for prolonged fevers associated with multiple other systemic and neurologic symptoms. He was found to have Lyme neuroborreliosis (NB), an early disseminated form of the disease, with specific findings of meningitis and polyradiculitis. As Lyme NB presents with such non-specific symptoms in pediatric patients, cases can easily be missed unless the diagnosis is high on the differential list. The resultant delay in management could lead to longstanding complications. This case serves as an important reminder of the illness presentation, especially for providers in high-risk regions.

## CASE REPORT

A six-year-old male with history of minimal vaccinations presented with his parents for evaluation of fatigue, headache, and vomiting that started after two weeks of a rash. Symptoms initially started three weeks prior with malaise, nausea, and tactile fevers. Upon resolution, a painful, non-pruritic, circular rash started on his right ankle and spread up his legs and back. He went to urgent care (UC), where he was diagnosed with viral hives and discharged home with five days of prednisone. The rash improved initially but returned, although no longer painful, after completion of the steroid burst.

Four days prior to arrival to the emergency department (ED), tactile fevers returned, his fatigue and rash worsened, and he developed a new-onset headache, vomiting (non-bloody, non-bilious), photophobia, diplopia, and myalgias. He went to another UC, was diagnosed with erythema multiforme and discharged on alternating acetaminophen and ibuprofen as well as loratadine. He slept constantly in the three days prior to presentation. The patient’s mother was also concerned because his gait was slow, and he appeared unsteady on his feet due to persistent leg pain. Several siblings lived at home with him, but none of them were sick. Mother denied known tick or other animal bites. He denied chest pain, shortness of breath, sore throat, cough, abdominal pain, and diarrhea.

**Image 1:**
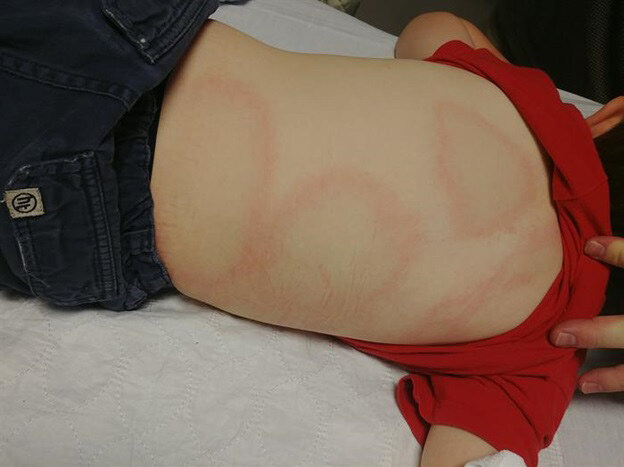
A 6-year-old male patient with an acute febrile illness presents with the depicted annular rash to his torso.

**Image 2:**
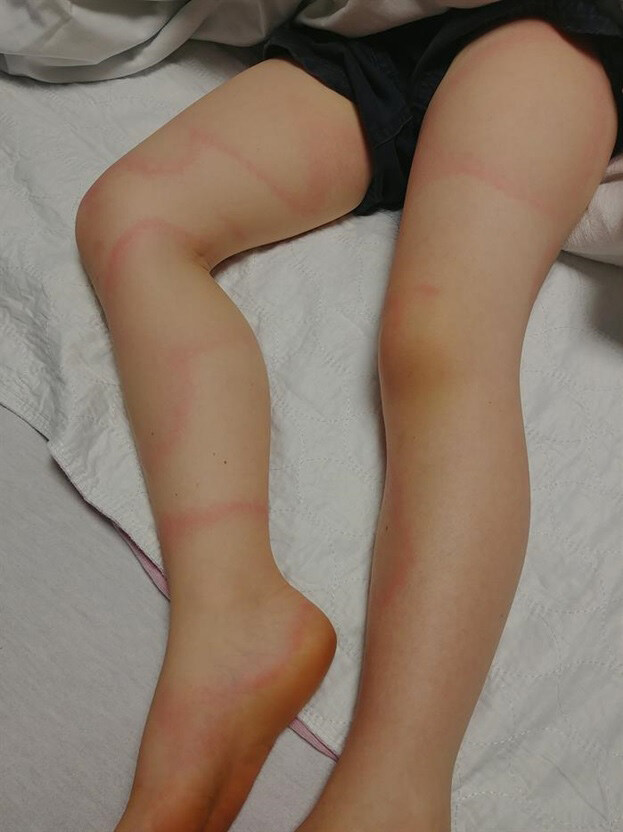
Image of the lower extremities demonstrating an annular rash similar to the rash present on the torso.

On examination, he was a very tired but nontoxic appearing male with normal vital signs for his age. His rash consisted of several large, annular, asymmetric, papular, blanchable lesions on his torso, legs, and back (Images 1, 2). There was no neck rigidity. Neurological examination was significant for decreased abduction of both eyes without complete paralysis. No other focal cranial deficits were noted. Neuromuscular exam demonstrated normal strength and sensation of all extremities. Deep tendon reflexes were 2+ throughout. Gait was slow and somewhat unsteady.

A head computed tomography (CT) was obtained and showed no intracranial lesions. Laboratory studies were drawn due to concern for possible infection and did not show significant abnormalities, apart from a slightly elevated erythrocyte sedimentation rate (Table). A lumbar puncture was then performed, which demonstrated a cerebrospinal fluid (CSF) pleocytosis (Table). With the overall benign lab evaluation and head CT combined with the CSF pleocytosis and rash resembling large erythema migrans (EM), there was concern for early disseminated Lyme disease complicated by meningitis. He was thus started on intravenous (IV) ceftriaxone and admitted to the hospital for further infectious disease and neurologic workup. Diagnosis was later confirmed by positive Lyme immunoglobulin M and immunoglobulin G antibodies of the blood and CSF. His hospitalization was complicated by sacral radiculopathy causing urinary retention, cranial nerve six palsy, and increased intracranial pressure with optic nerve swelling requiring administration of acetazolamide. He was discharged home on hospital day five with 16 days of oral doxycycline and had complete recovery on follow-up office visits.

## DISCUSSION

Lyme disease is caused by the spirochete *Borrelia burgdorferi* and is the most common tick-borne disease in the United States.^1^ The majority of cases occur in the Upper Midwest, Northeast, and Atlantic regions. Peak incidence of disease presentation is in the late spring and early summer, as people are more active outdoors in the warmer months.^1^

CPC-EM Capsule

What do we already know about this clinical entity?

*Lyme disease is divided into three stages (early localized, early disseminated, late) and can lead to long standing multisystem complications if left untreated*.

What makes this presentation of disease reportable?

*This patient presented with atypical neurologic symptoms from hematogenous spread of the disease, which ultimately led to a delay in the diagnosis*.

What is the major learning point?

*Evaluation of cerebrospinal fluid for* Borrelia burgdorferi *antibodies should be considered in patients presenting with viral-like illness and unusual neurologic symptoms*.

How might this improve emergency medicine practice?

*This case will hopefully prompt consideration of this diagnosis earlier in patients presenting with nonspecific viral and neurologic symptoms*.

Clinically, Lyme disease is divided into three stages: early localized; early disseminated; and late stage. The classic EM rash is the most common sign of early localized stage, occurring in at least 80% of patients usually 7–14 days after the tick bite.^1^ If left untreated, hematogenous dissemination can lead to involvement of multiple organ systems. Neuroborreliosis occurs when the spirochetes invade the nervous system, causing meningitis and inflammation of cranial and peripheral nerves.^3^,^4^ Late manifestations of the disease occur after six months and include Lyme arthritis and acrodermatitis chronic atrophicans.^1^

Key features of the history include known tick exposure or bite; however, most patients often do not recall a tick bite. The development of the single EM rash at the site of the tick bite with non-specific viral symptoms (fever, headache, fatigue, myalgias) is characteristic of early disease as well. Children with neuroborreliosis typically present with a facial nerve palsy and/or lymphocytic meningitis, but there has been documentation of other rare manifestations.^5^–^9^ In this case, the patient presented with symptoms of meningitis and ultimately developed involvement of multiple cranial and peripheral nerves.

The diagnosis of Lyme disease is often made clinically based on history of exposure and development of symptoms. If there is additional concern for NB, evaluation for antibodies, histopathologic and microbiologic evidence of *B. burgdorferi* has been recommended by the American Academy of Neurology.^10^ This typically includes detection of *Borrelia*-specific antibodies in CSF, which is mandatory for definitive diagnosis of NB.^3^,^5^ Direct detection of *B. burgdorferi* can be performed as well, but is seldom used due to its low sensitivity, long incubation period, and requirement of special culture media.^1^ Additional CSF findings include a lymphocytic pleocytosis, which appears similar to that of aseptic meningitis.^4^

There are multiple treatment options for treatment of pediatric Lyme disease, depending on the severity of infection and the age of the patient. In early uncomplicated Lyme disease, outpatient oral therapy with doxycycline is recommended.^3^,^4^,^11^ If the child is younger than eight years of age, amoxicillin is the drug of choice.^11^ Treatment for more severe infection such as NB consists of IV ceftriaxone preferably due to its favorable dosing schedule, but IV cefotaxime and IV penicillin G have been used successfully as well.^11^

The diagnosis of NB in children can be difficult because the symptoms may be nonspecific, leading to the possibility of a wide range of diseases and significant delay in diagnosis.^2^,^5^ Thus it is crucial for emergency physicians to consider evaluation of CSF for pleocytosis and *B. burgdorferi* antibodies in patients who present as in this case with viral-like illness and unusual neurological symptoms.

## CONCLUSION

Lyme disease classically presents with multiple, nonspecific symptoms and a characteristic rash. If left untreated, cardiac, neurologic, and rheumatologic complications can occur. Diagnosis of this illness can be difficult in the pediatric population due to symptom similarity to other diseases common throughout childhood. This is compounded when the presentation includes atypical neurologic symptoms. This case represents a unique case of neuroborreliosis presenting with partial bilateral abducens nerve palsy, peripheral nerve involvement, and lymphocytic meningitis. This case emphasizes the importance of considering Lyme disease in pediatric patients who present with rash, viral-like illness, and nonspecific neurologic symptoms.

## Figures and Tables

**Table:** Laboratory data of six-year-old child diagnosed with neuroborreliosis.

Variable	Vaalue	Reference Range
Sodium (mmol/L)	134	135–145
Potassium (mmol/L)	4.4	3.7–5.6
Chloride (mmol/L)	98	95–106
Carbon Dioxide (mmol/L)	22	18–27
Glucose (mg/dL)	82	60–115
Urea Nitrogen (mg/dL)	12	5–18
Creatinine (mg/dL)	0.41	0.10–0.60
Calcium (mg/dL)	9.0	8.0–10.5
Magnesium (mg/dL)	2.0	1.5–2.4
Phosphorous (mg/dL)	4.9	4.1–5.9
WBC (10^6^/uL)	11.3	5.0–14.5
Neutrophils (%)	84.4	36.0–72.0
Lymphocytes (%)	11.3	27.0–57.0
Monocytes (%)	3.9	2.0–8.0
Eosinophils (%)	0.2	1.0–4.0
Basophils (%)	0.2	0.0–1.0
RBC (10^6^/mL)	4.67	4.0–5.2
Hemoglobin (g/dL)	12.3	11.5–15.5
Hematocrit (%)	35.6	35–45
Platelet count (10^3^/uL)	293	140–440
Mononucleosis Screen	Negative	Negative
Sedimentation Rate (mm/h)	45	0–13
C-reactive protein (mg/dL)	1.1	< 1.2
Cell count, CSF (cells/uL)	22	None
Color	Clear, Colorless	None
RBC, CSF fluid (/mm^3^)	0	None
WBC, CSF fluid (/mm^3^)	22	None
PMNc, CSF fluid (%)	28	None
Lymphocytes, CSF fluid (%)	50	None
Monocyte/macrophage, CSF fluid (%)	22	None
Gram stain	No organisms seen	None
Glucose, CSF (mg/dL)	45	40–70
Protein, CSF (mg/dL)	18	15–45

*mmol*, millimole; *L*, liter; *mg*, milligram; *dL*, deciliter; *WBC*, white blood cell; *uL*, microliter; *%*, percent; *RBC*, red blood cell; *mL*, milliliter; *g*, gram; *uL*, microliter; *mm*, millimeter; *h*, hour; *CSF*, cerebrospinal fluid; *PMNc*, polymorphonuclear cell.

## References

[1] Marques AR (2015). Lyme Neuroborreliosis. Continuum (Minneap Minn).

[2] Esposito S, Bosis S, Sabatini C (2013). *Borrelia burgdorferi* infection and Lyme disease in children. Int J Infect Dis.

[3] Skogman BH, Croner S, Nordwall M (2008). Lyme neuroborreliosis in children: a prospective study of clinical features, prognosis, and outcome. Pediatr Infect Dis J.

[4] Pachner AR (1995). Early disseminated Lyme disease: Lyme meningitis. Am J Med.

[5] Baumann M, Birnbacher R, Koch J (2010). Uncommon manifestations of neuroborreliosis in children. Eur J Paediatr Neuro.

[6] Ewers EC, Dennison DH, Stagliano DR (2015). A unique case of adolescent neuroborreliosis presenting with multiple cranial neuritis and cochlear inflammation on magnetic resonance imaging. Pediatr Neurol.

[7] Roaldsnes E, Eikeland R, Berild D (2017). Lyme neuroborreliosis in cases of non-specific neurological symptoms. Tidsskr Nor Laegeforen.

[8] Miller MM, Müllegger RR, Spork KD (1991). Lyme borreliosis of central nervous system (CNS) in children: a diagnostic challenge. Infection.

[9] Vukelic D, Bozinovic D, Morovic M (2000). Opsoclonus-myoclonus syndrome in a child with neuroborreliosis. J Infect.

[10] Halperin JJ, Logigian EL, Finkel MF (1996). Practice parameters for the diagnosis of patients with nervous system Lyme borreliosis (Lyme disease). Neurology.

[11] Wormser GP, Dattwyler RJ, Shapiro ED (2006). The clinical assessment, treatment, and prevention of Lyme disease, human granulocytic anaplasmosis, and babesiosis: clinical practice guidelines by the Infectious Diseases Society of America. Clin Infect Dis.

